# Coordination of m^6^A mRNA methylation and gene transcriptome in rice response to cadmium stress

**DOI:** 10.1186/s12284-021-00502-y

**Published:** 2021-07-05

**Authors:** Qin Cheng, Peng Wang, Guangliang Wu, Yanning Wang, Jingai Tan, Caijing Li, Xiangyu Zhang, Shilei Liu, Shiying Huang, Tao Huang, Mengmeng Yang, Haohua He, Jianmin Bian

**Affiliations:** 1 Key Laboratory of Crop Physiology, Ecology and Genetic Breeding, Ministry of Education, Jiangxi Agricultural University, 330045 Nanchang, China; 2 College of Agronomy, Jiangxi Agricultural University, 330045 Nanchang, China

**Keywords:** N^6^-methyladenosine, Posttranscriptional regulation, Rice, Cadmium stress, Seedling

## Abstract

N^6^-methyladenosine (m^6^A) is the most prevalent internal modification present in the mRNAs of all higher eukaryotes. However, the role of the m^6^A methylomes in rice is still poorly understood. With the development of the MeRIP-seq technique, the in-depth identification of mRNAs with m^6^A modification has become feasible. A study suggested that m^6^A modification is crucial for posttranscriptional regulation related to Cd^2+^-induced malignant transformation, but the association between m^6^A modification in plants and Cd tolerance has not been reported. We investigated the m^6^A methylomes in the roots of a cadmium (Cd)-treated group and compared them with the roots in the control (CK) group by m^6^A sequencing of cv. 9311 and cv. Nipponbare (NIP) plants. The results indicated that Cd leads to an altered modification profile in 3,406 differential m^6^A peaks in cv. 9311 and 2,065 differential m^6^A peaks in cv. NIP. KEGG pathway analysis of the genes with differentially modified m^6^A peaks indicated that the “phenylalanine”, “tyrosine and tryptophan biosynthesis”, “glycine”, “adherens junctions”, “glycerophospholipid metabolism” and “threonine metabolism” signalling pathways may be associated with the abnormal root development of cv. 9311 rice due to exposure to Cd. The “arginine”, “proline metabolism”, “glycerolipid”, and “protein processing in endoplasmic reticulum” metabolism pathways were significantly enriched in genes with differentially modified m^6^A peaks in cv. NIP. Unlike that in *Arabidopsis*, the m^6^A-modified nucleotide position on mRNAs (m^6^A peak) distribution in rice exhibited a preference towards both the stop codon and 3′ untranslated regions (3′ UTRs). These findings provide a resource for plant RNA epitranscriptomic studies and further increase our knowledge on the function of m^6^A modification in RNA in plants.

**Supplementary Information:**

The online version contains supplementary material available at 10.1186/s12284-021-00502-y.

## Introduction

m^6^A is one of the most important internal modifications present in the mRNAs of many eukaryotic species, including yeast, plants (Wei et al. 2018), flies (Lence et al. 2016), and mammals (Yang et al. 2018; De et al. 2019). In mammals, this modification is dynamic and plays important roles in the regulation of mRNA metabolism and processing (Duan et al. 2017), including alternative splicing, exportation, stability, translation, and microRNA maturation (Yang et al. 2018; Shen et al. 2016). The functions of m^6^A on RNA are determined by the dynamic interplay between a conserved set of proteins called writers, erasers and readers (Meyer and Jaffrey 2017). methyltransferase-like 3(METTL3) is the first m^6^A methyltransferase to be identified in mammals and is highly conserved in plants and mammals (Yao et al. 2019). methyltransferase-like 14(METTL14) is the second most active m^6^A methyltransferase enzyme in humans to catalyse m^6^A RNA methylation and is highly homologous to METTL3. Other components, such as RNA binding motif protein 15 (RBM15), Cbl photo oncogene like 1(HAKAI), and zinc finger CCCH domain-containing protein 13(ZC3H13), have been shown to directly regulate RNA modification.

In plants, most of the progress in elucidating the methylation mechanism and function of m^6^A has been made in *Arabidopsis* (Zhang et al. 2019). In *Arabidopsis*, mRNA adenosine methylase (MTA) is homologous to METTL3, MTB to METTL14, and FKBP12 interacting protein 37 (FIP37) to Wilms’ tumour1-associating protein (WTAP). Recent studies have shown that ALKBH10B-mediated mRNA demethylation influences floral transition by affecting the stability of target transcripts (Duan et al. 2017). It has been reported that the cytoplasmic *Arabidopsis* for YT521-B Homology (YTH) domain proteins EVOLUTIONARILY CONSERVED C-TERMINAL REGION2/3 (ECT2/3) are required for the correct timing of leaf formation and normal leaf morphology (Arribas-Hernández et al. 2018). In addition, a study showed that AtFIP37 plays an indispensable role in determining the fate of stem cells in *Arabidopsis* (Shen et al. 2016). Taken together, these studies indicate that m^6^A has unique functions during the life cycle of *Arabidopsis*. There is increasing evidence that m^6^A is also involved in regulating responses to various abiotic and biological stresses (Yue et al. 2019). Recent studies have shown that m^6^A modifications are involved in the regulation of responses to salt stress; for example, in *Arabidopsis thaliana*, m^6^A generally acts as a stabilizing mark through the inhibition of site-specific cleavage in plant transcriptomes, and this mechanism is required for the proper regulation of the salt stress-responsive transcriptome (Anderson et al. 2018). In sweet sorghum, m^6^A modification regulates mRNA abundance by regulating the stability of salt-tolerant transcripts (Zheng et al. 2021). A recent study provided a comprehensive reference map of gene activity through multiomics analysis, revealing that the m^6^A signalling pathway is critical in Cd^2+^ carcinogenesis (Wu et al. 2021), but the association between m^6^A modification in plants and Cd tolerance has not been reported. Different cellular pressures can lead to a redistribution of m^6^A within the transcriptome, resulting in an increase in the number of mRNAs with 5′UTR m^6^A (Meyer et al. 2015). The m^6^A pattern is dynamic, and 5–30 % of m^6^A peaks are altered by ultraviolet light, heat shock, or interferon-gamma, thereby affecting gene expression and splicing (Meyer et al. 2012). Studies have shown that m^6^A can dynamically regulate the response of cells to abiotic stresses, including heat shock, ultraviolet light, hypoxia and oxidative stress (Parker et al. 2020).

Despite its importance, much of the original work on m^6^A has focused on humans and model animals such as mice, while few studies have explored its role in rice. One study revealed for the first time that OsFIP plays an indispensable role in rice early sporogenesis (Yao et al. 2019). Rice is one of the most important food crops in China and an important monocotyledonous model organism. Cd accumulation in rice grains poses a serious threat to human health and Cd is a widespread, detrimental, heavy metal pollutant that poses potential chronic toxicity to living organisms (Tan et al. 2017; Cao et al. 2019). In plants, the most obvious effect of Cd toxicity is a reduction in plant growth related to an inhibition of photosynthesis, respiration and nitrogen metabolism, as well as to a reduction in water and nutrient uptake (Santos et al. 2012). A study showed that many genes were involved in the stress response, including metal transport and transcription factors, and most of the DNA methylation-modified genes were transcriptionally altered under Cd stress (Feng et al.^2016^). Moreover, hypomethylation is associated with gene expression during Cd detoxification and accumulation in rice, and the newly identified mechanism for the enhanced expression of the Cd resistance gene *OsCTF* may help develop engineered crops (Feng et al. 2020). Recently, a study indicated that Cd exposure causes dramatic changes in the cytosine methylation status of the plant genome, thus affecting the expression of many genes that are vital for plant growth and are involved in the Cd stress response (Xin et al. 2019). The *japonica* group cultivar cv. NIP and *indica* group cultivar cv. 9311 are the two main cultivated varieties and are common parental lines used for breeding in Asia. The complexity of rice Cd transport and accumulation indicates the need to understand what is responsible for the Cd accumulation divergence between *indica* and *japonica* rice subspecies.

Thus, in this work, we aimed to obtain further understanding of the effects of Cd on rice roots in terms of m^6^A methylation in mRNA. We report the m^6^A sequencing profiling of two accessions of rice, *indica* rice cv. 9311 and *japonica* rice cv. NIP. To investigate the different Cd response mechanisms in different cultivars, we studied the enriched metabolic pathways of the differential m^6^A modification peaks. Collectively, our data will constitute a comprehensive picture of m^6^A methylation in mRNA in rice roots and provide the basis for future studies of its function and biological significance in rice.

## Results

### Rice root growth was affected by Cd stress

**Fig. 1:**
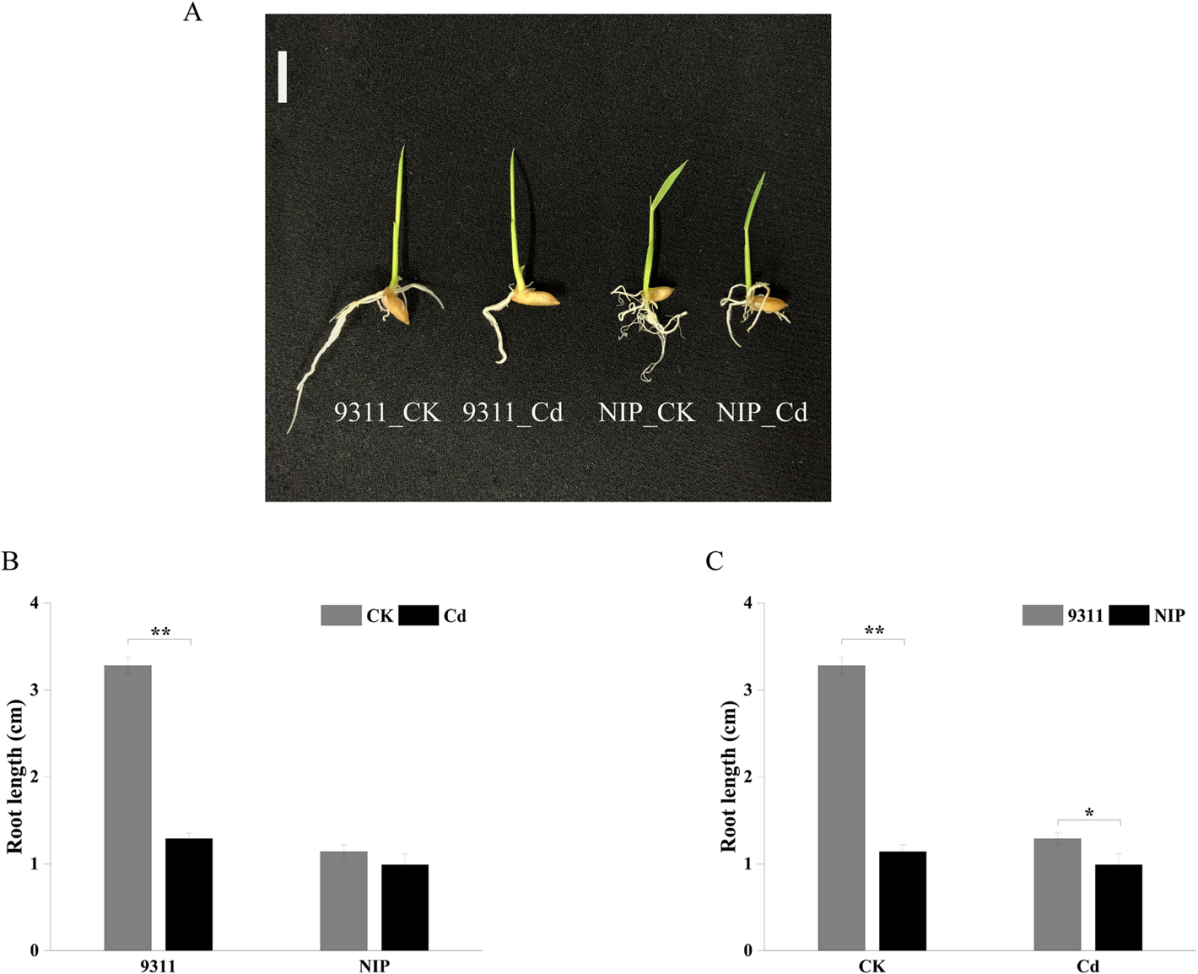
Phenotypes of cv. 9311 and cv. NIP under CK (control) and Cd (cadmium)groups. (**A**) The 3-day-old seedlings of cv. 9311 and cv. NIP under control condition and Cd stress conditions. (**B**) and (**C**) Comparison of root length, in 3-day-old seedlings of cv. 9311 and cv. NIP under under control condition and Cd stress conditions, respectively. Data are presented as means ± SE. *n* = 15. Statistical analysis was conducted using the Student’s t-test. *, *P*-value < 0.05; **, *P*-value < 0.01; ***, *P* value < 0.001. scale bar = 1 cm

Cd stress induced phenotypic variations in rice seedlings. The root lengths of cv. 9311 and cv. NIP were shortened in the Cd group compared with the CK group (CK vs. Cd; *Student’s t-test*, p-value < 0.01 or 0.01 < p-value < 0.05) (Fig. 1 A and 1B) (the concentration of Cd was 50 µM in the Cd group). Interestingly, we observed that cv. 9311 was more sensitive to cadmium than cv. NIP, and the root length of cv. 9311 was significantly longer than that of cv. NIP under the control conditions. In cv. 9311, the average length of rice roots in the CK group was 3.2 cm, while in the Cd group, it was 1.2 cm, and the average length of rice shoots in the CK group was 3.2 cm, while in the Cd group, it was 3.0 cm (Supplementary Fig. S1A). In cv. NIP, the average length of rice roots in the CK group was 1.1 cm, while in the Cd group, it was 0.9 cm, the average length of rice shoots in the CK group was 2.8 cm, while in the Cd group, it was 2.6 cm (Supplementary Fig. S1B). The root length of cv. 9311 was significantly longer than that of cv. NIP in the CK group (cv. 9311_CK vs. cv. NIP_CK; *Student’s t-test*, *p*-value < 0.01), while there were no significant differences between the two genotypes in the Cd group at the same stages (cv. 9311_Cd vs. cv. NIP_ Cd; *Student’s t-test*, *p*-value > 0.05) (Fig. 1 C). These results inspired us to investigate how Cd stress changes rice roots in these two rice cultivars.

### Generation of m^6^A methylation profiles for rice roots

**Fig. 2:**
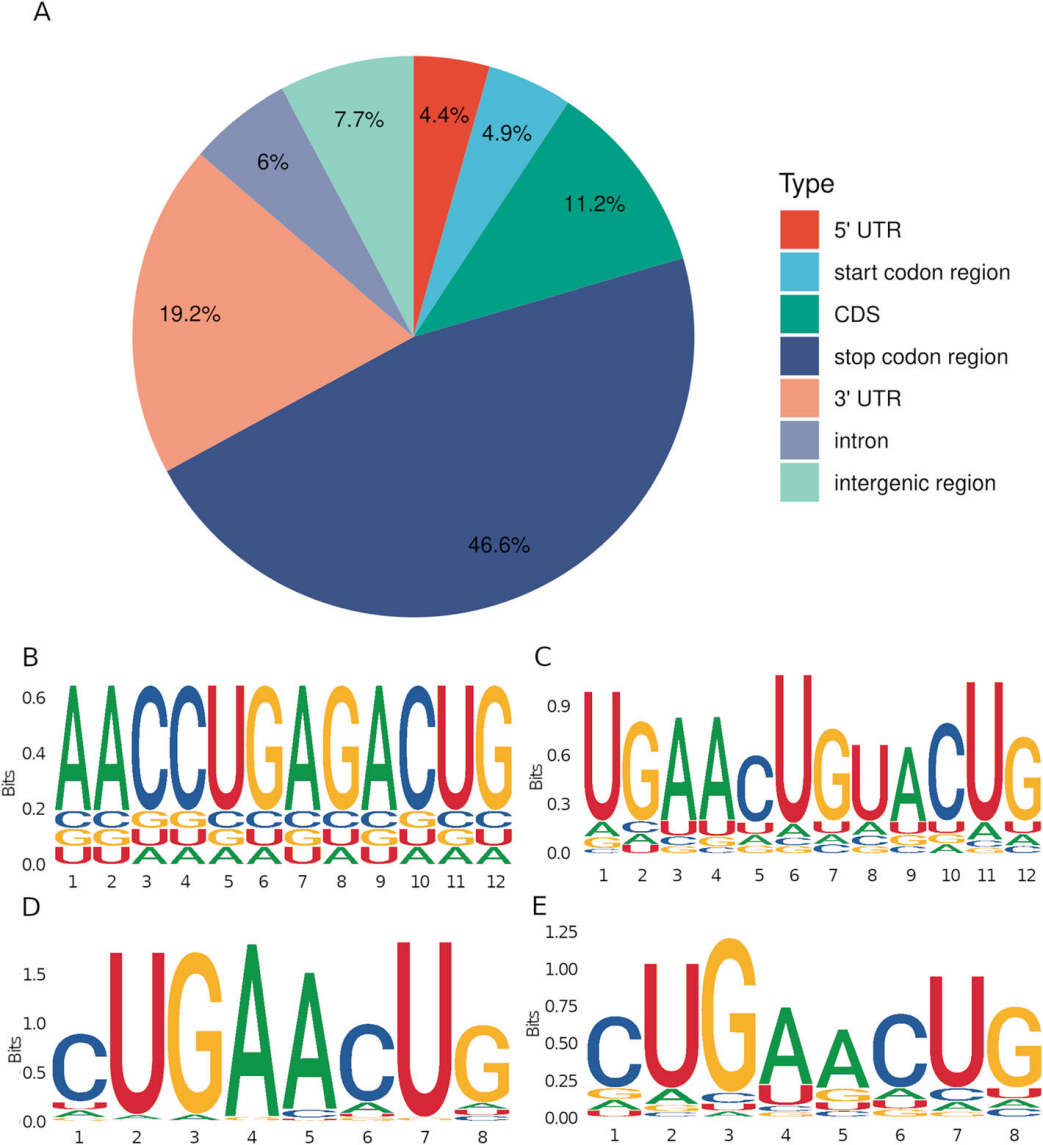
Overview of m^6^A methylome in rice. (**A**) Annotation of identified m^6^A high confident peaks (**B**)**-**(**E**) canonical RRACH and motif URUAY motif in four samples

To obtain the transcriptome-wide m^6^A map in rice seedlings, a series of m^6^A-immunoprecipitation (IP) and matched input (non-IP control) libraries were constructed and sequenced (Supplementary Table S1). Clean reads were obtained, resulting in 59–77 million clean reads for each library. A total of 8,972, 8,239, 8,706 and 7,813 peaks were present in at least 2 out of the 3 biological replicates for cv. 9311 and cv. NIP in the CK and Cd groups, respectively (Supplementary Table S2). These m^6^A peaks from different experimental conditions were further merged into a unique set of 10,735 m^6^A peaks, 92.26 % (9,904) of which were present in the genic regions of 9,802 genes (minimum overlap was 100 bp), accounting for an average of 1.01 m^6^A peaks within transcription units from each gene. We randomly selected seven m^6^A-methylated genes and validated their m^6^A modification using m^6^A reverse transcription quantitative PCR (RT-qPCR) (Supplementary Fig. S2). These 10,735 m^6^A peaks in rice were enriched in the stop codon region (46.6 % of m^6^A peaks), followed by the 3’UTR (19.2 %) and coding region (11.2 %) (Fig. 2 A). Similar distribution patterns of m^6^A peaks were also observed in a separate analysis of m^6^A-seq data from each cv. 9311 or cv. NIP group (Supplementary Fig. S3). The distribution pattern of m^6^A peaks in rice is similar to that observed in maize (Miao et al. 2020) and *Arabidopsis* (Shen et al. 2016).

As expected, we also observed that 10,730 m^6^A peaks (99.95 %) contained the canonical motif RRACH (where R represents A/G, A is m^6^A, and H represents A/C/U) in rice and 10,367 m^6^A peaks (96.57 %) contained the canonical motif URUAY (where Y represents C/U; Fig. 2B-E), which could also be detected in the m^6^A peaks from each replicate sample.

### m^6^A methylation is affected by Cd stress in cv. NIP and cv. 9311

**Fig. 3:**
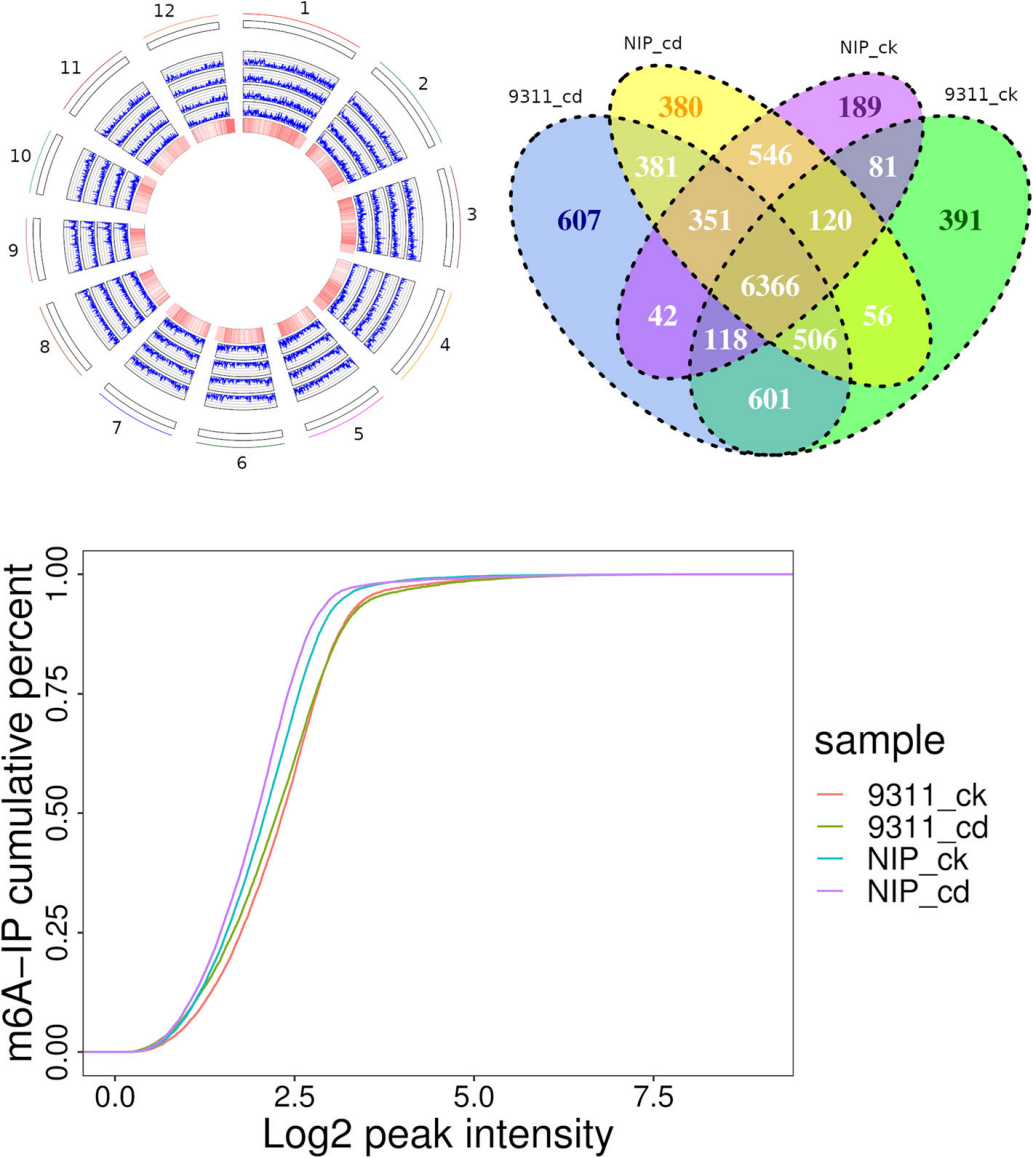
The m^6^A methylome changes under Cd stress in rice. (**A**) Genomic distribution of high confidience peaks (hcpeaks) in four groups. The reference genome is split into 100 kb bins and the frequencies of genes or peaks located in each 100 kb bin was counted and plot as line. The tracks are “gene (heatmap)”, “peaks in cv. 9311_cd”, “peaks in cv. 9311_ck”, “peaks in cv. NIP_cd” and “peaks in cv. NIP_ck” from inside outwards. (**B**) Comparison of high confidence peaks (hcpeaks) in 4 groups. (**C**) Cumulative distribution function of log2 peak intensity of m^6^A-modified sites under CK and Cd groups in cv. 9311 and cv. NIP

**Fig. 4:**
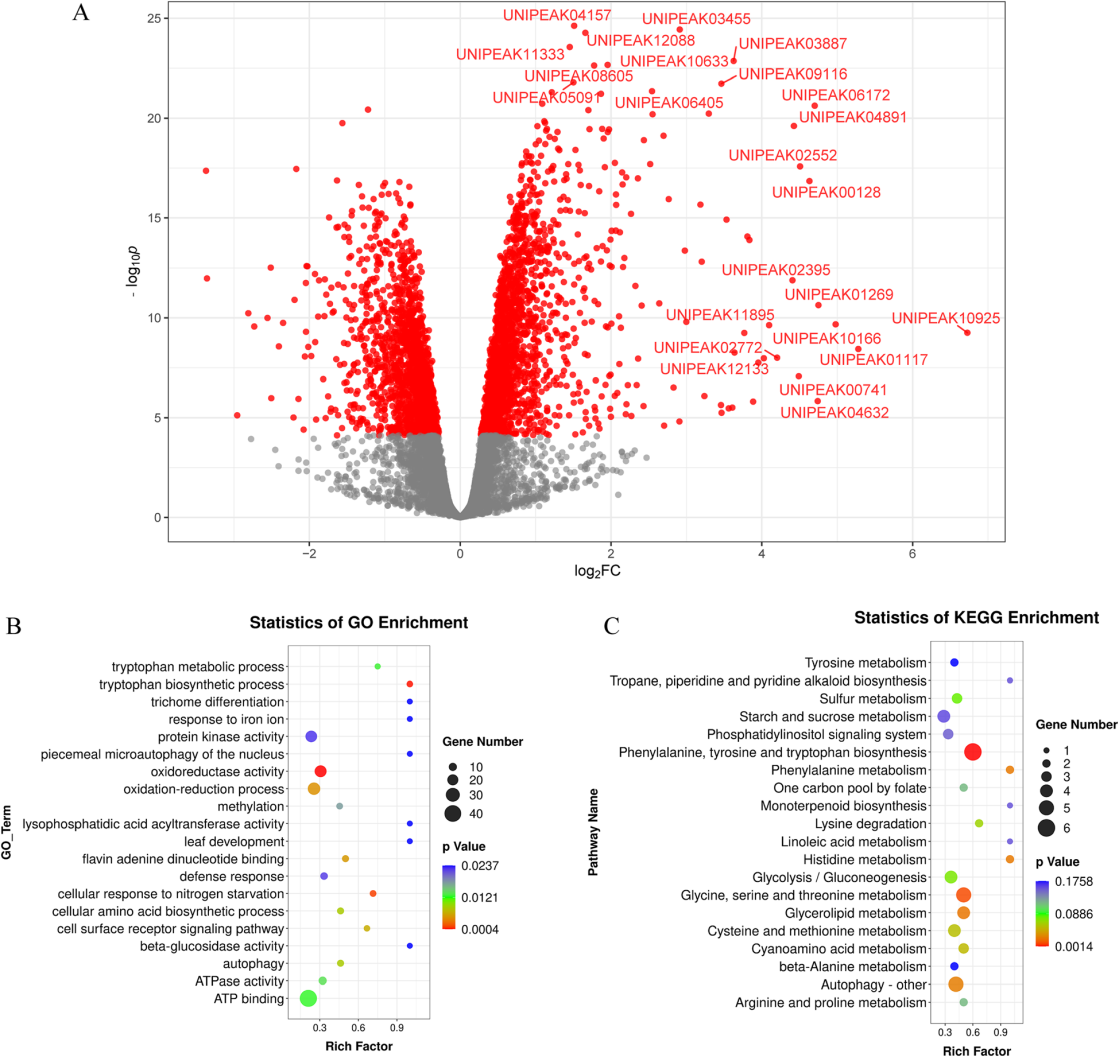
Gene ontology and Kyoto Encyclopedia of Genes and Genomes analyses of coding genes containing differentially enriched m^6^A hcpeaks in cv. 9311_CK vs. cv. 9311_Cd. (**A**) Volcano plot showing logFC against statistical significance. Hcpeaks with FDR < 0.05 are coloured red with select points at the extremities of the plot labelled for easier visualisation (top 10 DE peaks or logFC < -4 or logFC > 4) (**B**) Major gene ontology terms were significantly enriched for these genes. (**C**) Major Kyoto Encyclopedia terms were significantly enriched for these genes

Using data from these two cultivars, we investigated whether and to what extent Cd stress changes the m^6^A methylation of genes in rice genotypes with different tolerances to Cd stress. First, we examined the genomic distribution of m^6^A peaks in rice roots under different experimental conditions (Fig. 3 A). At the genome level, the 10,735 peaks were unevenly distributed across each chromosome. The majority of high confidence peaks (hcpeaks) were present in all four different experimental conditions (Fig. 3B). The saturation curve showed that the RNA methylation levels of the Cd group were lower than those of the CK group for both cv. 9311 and cv. NIP (Fig. 3 C). We further compared all peaks in the CK and Cd groups across different rice cultivars (cv. 9311 and cv. NIP). In cv. 9311, 7,591 hcpeaks within mRNAs (∼79 % of all peaks in the CK and Cd groups) overlapped between the CK and Cd groups, and 3,406 hcpeaks were identified as significantly differentially enriched hcpeaks in the Cd group compared to the CK group (FDR < 0.05) (Fig. 4 A). Gene Ontology (GO) enrichment analysis of the genes in these differentially enriched m^6^A hcpeaks showed that “ATP binding”, “protein kinase activity”, “oxidoreductase activity and “oxidation − reduction process were enriched (Fig. 4B). KEGG pathway analysis of the genes in these differentially enriched m^6^A hcpeaks showed that “phenylalanine”, “tyrosine and tryptophan biosynthesis”; “glycine, serine and threonine metabolism”; and “cysteine” and “methionine metabolism” pathways were enriched (Fig. 4 C).

**Fig. 5:**
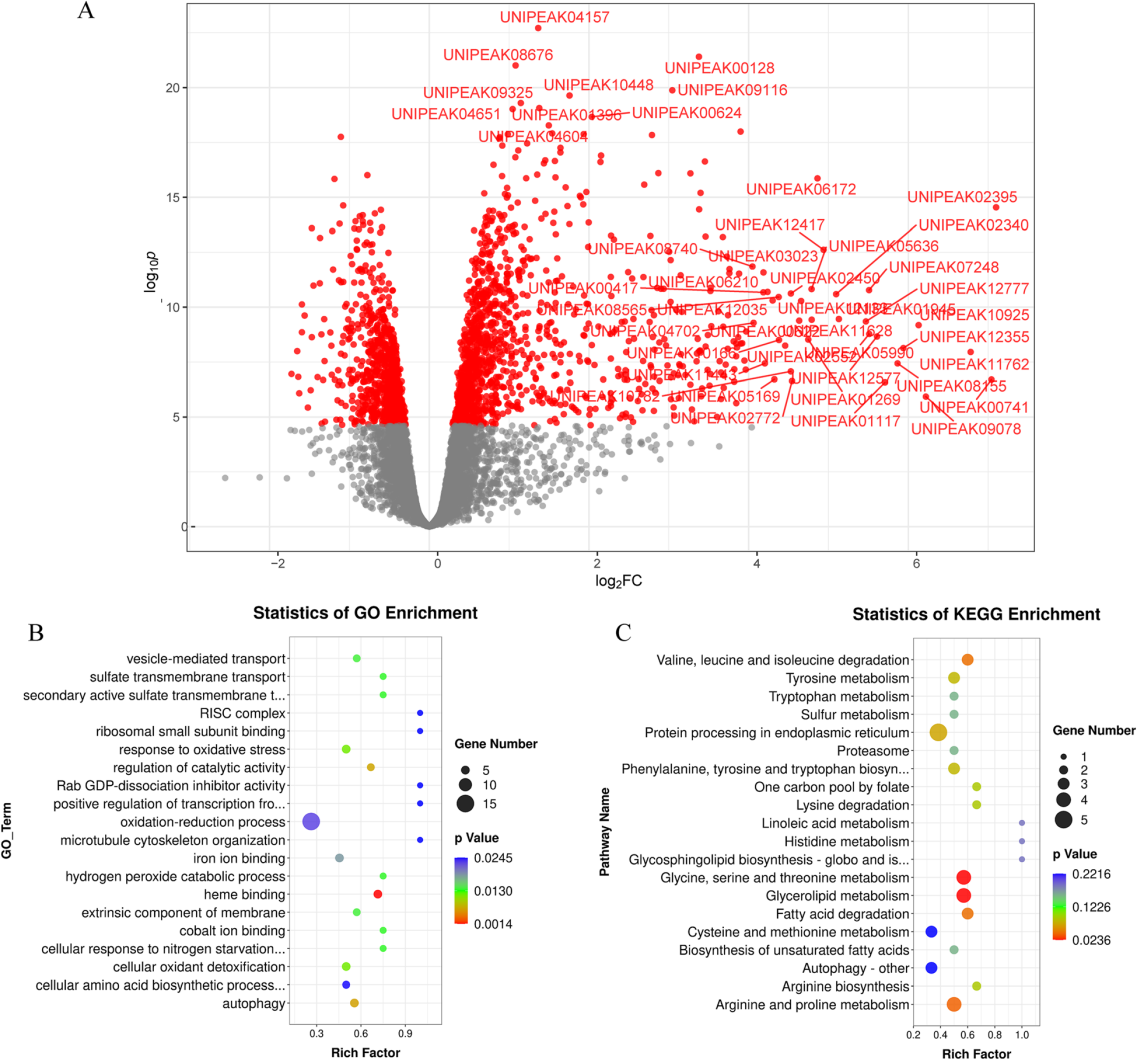
Gene ontology and Kyoto Encyclopedia of Genes and Genomes analyses of coding genes containing differentially enriched m^6^A hcpeaks in cv. NIP_CK vs. cv. NIP_Cd. (**A**) Volcano plot showing logFC against statistical significance. Hcpeaks with FDR < 0.05 are coloured red with select points at the extremities of the plot labelled for easier visualisation (top 10 DE peaks or logFC < -4 or logFC > 4) (**B**) Major gene ontology terms were significantly enriched for these genes. (**C**) Major Kyoto Encyclopedia terms were significantly enriched for these genes

In cv. NIP, 7,383 hcpeaks within mRNAs (∼80.8 % of all peaks in the CK and Cd groups) overlapped between the CK and Cd groups, and 2,065 hcpeaks were identified as differentially methylated peaks (DMPs) (FDR < 0.05) (Fig. 5 A). GO terms including “transferase activity”, “transferring glycosyl groups”, “defence response to bacterium” and “cell surface receptor signalling” were particularly enriched in the genes overlapping with these differentially enriched m^6^A hcpeaks (Fig. 5B). With respect to KEGG pathways, “arginine and proline metabolism”, “protein processing in endoplasmic reticulum” and “glycerolipid metabolism” pathways were significantly enriched in the genes overlapping with these differentially enriched m^6^A hcpeaks (Fig. 5 C).

**Fig. 6:**
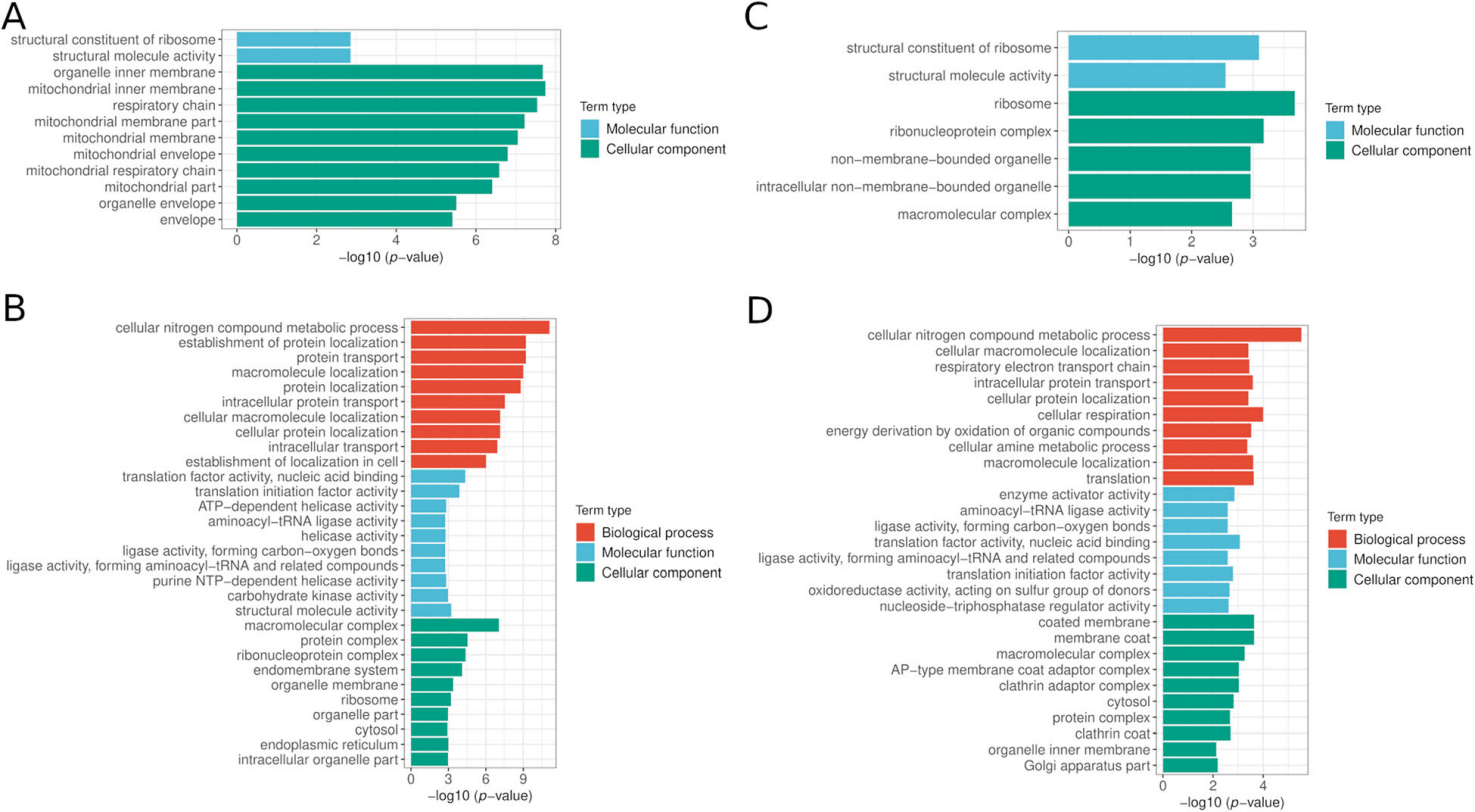
Functional characteristics of differentially methylated peaks (DMPs) in the context of genic location in cv. 9311 and cv. NIP. (**A**) top 10 over-represented GO term in each category at their 5’-UTR in cv. 9311 (**B**) top 10 over-represented GO term in each category at their 3’-UTR in cv. 9311(**C**) top 10 over-represented GO term in each category at their 5’-UTR in cv. NIP(**D**) top 10 over-represented GO term in each category at their 3’-UTR in cv. NIP

To investigate whether genes with m^6^A methylation at different genic regions have different functions in rice, we performed GO and KEGG functional overrepresentation analysis for genes with m^6^A methylation at the 5’-UTR or 3’-UTR. We observed that in cv. 9311, the GO terms “mitochondrial inner membrane” and “organelle inner membrane”, which are in the “cellular component” category, were specifically enriched in genes with DMPs within the 5’-UTR (Fig. 6 A), whereas the GO terms “cellular nitrogen compound metabolic process” and “establishment of protein localization” were specifically enriched in genes within DMPs near the 3’-UTR (Fig. 6B). In cv. NIP, the GO terms “ribosome” and “structural constituent of ribosome” were specifically enriched in genes with DMPs within the 5’-UTR (Fig. 6 C), whereas the GO terms “cellular nitrogen compound metabolic process” and “cellular macromolecule localization” were specifically enriched in genes within significantly differentially enriched hcpeaks near the 3’-UTR (Fig. 6D). These results revealed that genes containing significantly differentially enriched hcpeaks in specific genic locations play roles in distinct biological processes in cv. 9311 and cv. NIP.

Our study suggests that the number and extent of m^6^A modifications on the transcripts of Cd resistance genes may be important factors for determining and assessing the Cd tolerance of crops.

### Conjoint analysis of genes with differential m^6^A peaks and differential expression

**Fig. 7:**
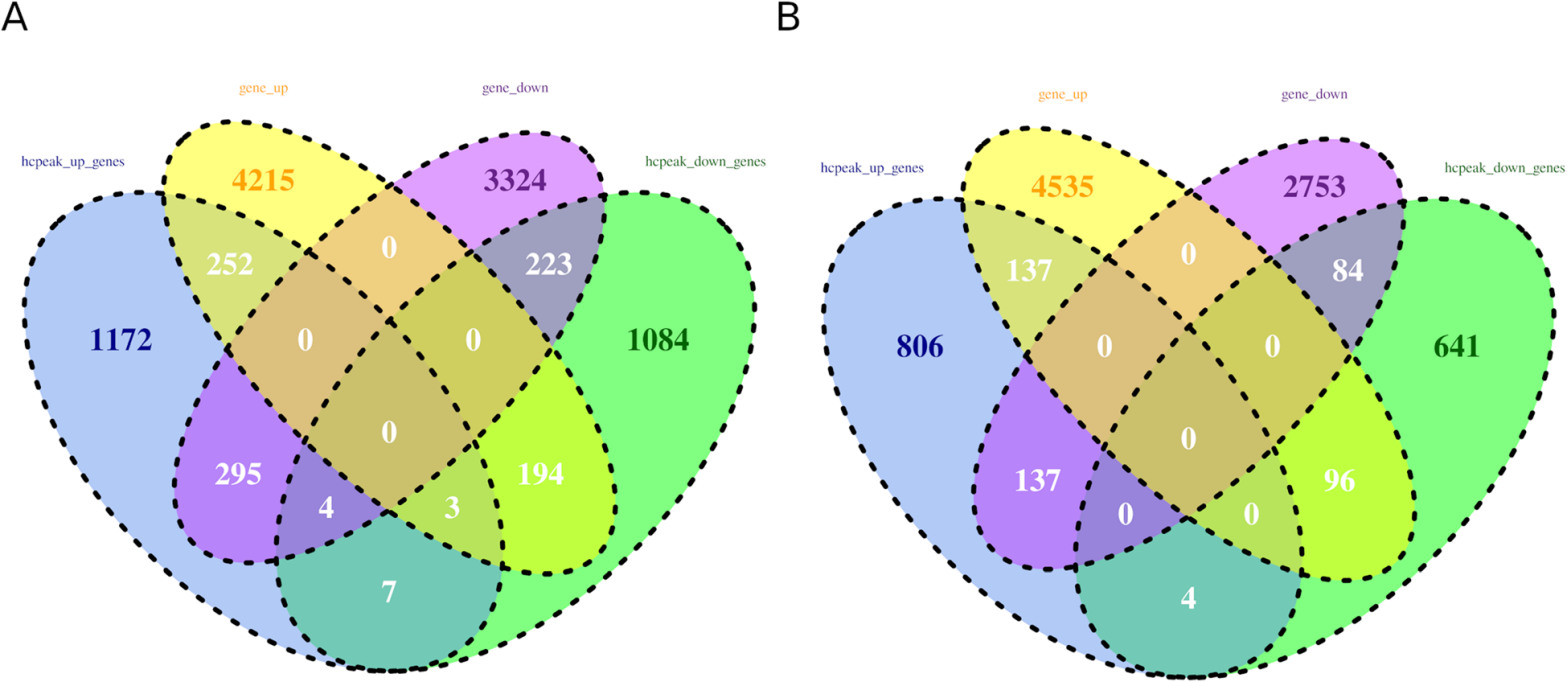
Compare the differential hcpeaks and DE genes. (**A**) comparison of genes overlapping with differential hcpeaks and DE genes in cv. 9311_cd vs. cv. 9311_ck (**B**) comparison of genes overlapping with differential hcpeaks and DE genes in cv. NIP _ cd vs. cv. NIP _ck

Differentially expressed genes (DEGs) were identified by comparing samples of the same rice cultivar under different conditions and different rice cultivars (cv. 9311 and cv. NIP) under the same conditions; in total, two comparison groups (cv. 9311_Cd vs. cv. 9311_CK and cv. NIP_ Cd vs. cv. NIP_CK) were obtained. A total of 8,510 DEGs were identified as differentially expressed genes (FDR < 0.05) in cv. 9311_Cd vs. cv. 9311_CK, and among them, 4,664 were upregulated and 3,846 were downregulated. According to the peak differential analysis, 3,406 significantly differential (FDR < 0.05) peaks were identified in cv. 9311_Cd compared to cv. 9311_CK; among them, 1,810 overlapping with 1,733 genes were upregulated and 1,596 overlapping with 1,515 genes were downregulated. The comparison of the overlapping differential peaks and DEGs in the comparison of “cv. 9311_Cd vs. cv. 9311_CK” is shown in Fig. 7 A. At the same time, 7,742 significantly DEGs (FDR < 0.05) were identified in cv. NIP_ Cd compared to cv. NIP_CK, and among them, 4,768 were upregulated and 2,974 were downregulated. According to differential peak analysis, 2,065 significantly differential (FDR < 0.05) peaks were identified in cv. NIP _Cd compared to cv. NIP_CK, and among them, 1,191 overlapping with 1,084 genes were upregulated and 874 overlapping with 825 genes were downregulated. The comparison of the overlapping differential peaks and DEGs in the comparison of “cv. NIP_ Cd vs. cv. NIP_ CK” is shown in Fig. 7B.

In addition, differential m^6^A peaks and DEGs between the two different rice cultivars (cv. 9311_Cd vs. cv. NIP_ Cd and cv. 9311_CK vs. cv. NIP_CK) were also analysed. A total of 6,658 DEGs were identified as differentially expressed genes (FDR < 0.05) in cv. 9311_Cd vs. cv. NIP_Cd, among which 2,389 were upregulated and 4,269 were downregulated. According to differential peak analysis, 3,330 significantly differential (FDR < 0.05) hcpeaks were identified in cv. 9311_Cd compared to cv. NIP_ Cd, and among them, 1,598 overlapping with 1,401 genes were upregulated and 1,732 overlapping with 1,550 genes were downregulated. The comparison of the overlapping differential peaks and DEGs in the comparison of “cv. 9311_Cd vs. cv. NIP_ Cd” is shown in Supplementary Fig. S4A. At the same time, 6,992 significantly DEGs (FDR < 0.05) were identified in cv. 9311_CK compared to cv. NIP_CK, and among them, 2,985 were upregulated and 4,007 were downregulated. A total of 3,499 significantly differential (FDR < 0.05) hcpeaks were identified in cv. 9311_CK compared to cv. NIP_CK, and among them, 1,720 overlapping with 1,482 genes were upregulated and 1,779 overlapping with 1,594 were downregulated. The comparison of the overlapping differential peaks and DEGs in the comparison of “cv. 9311_CK vs. cv. NIP_CK” is shown in Supplementary Fig. S4B.

This result indicated that not only the cultivar but also the treatment affected the gene expression level and m^6^A mRNA methylation level. Moreover, the number of differential peaks and DEGs in cv. NIP_ Cd vs. cv. NIP_CK was less than that in cv. 9311_Cd vs. cv. 9311_CK. These results further suggest that cv. 9311 is more sensitive to Cd than cv. NIP.

### Combined analysis of differential m^6^A methylation in cv. NIP and cv. 9311

**Fig. 8:**
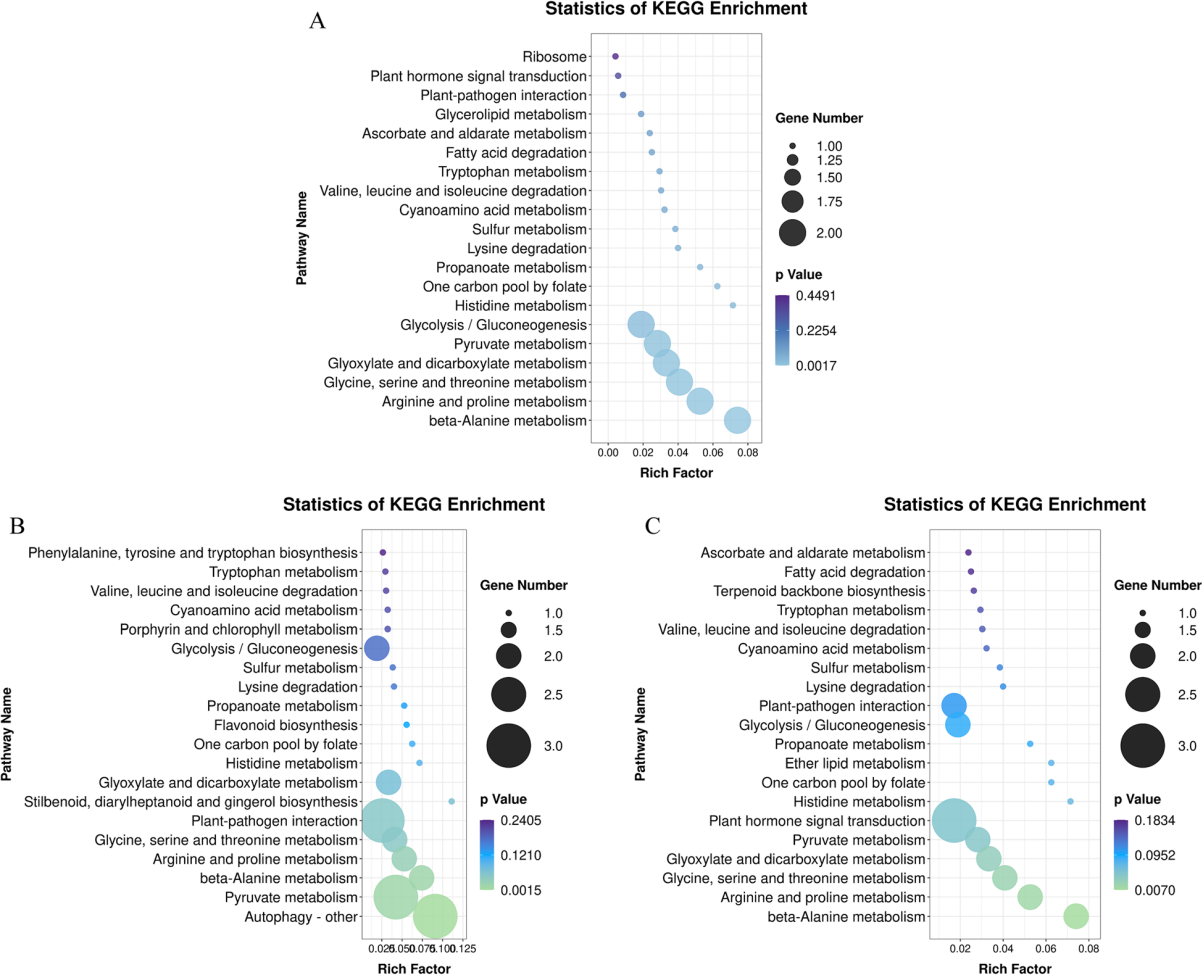
The KEGG terms of differential m^6^A methylation genes in cv. NIP and cv. 9311. (**A**) the KEGG terms of common genes with differential m^6^A methylation in *indica* and *japonica* under cadmium stress (**B**) the KEGG terms of unique genes with differential m^6^A peaks in *indica* (**C**) the KEGG terms of unique genes with differential m^6^A peaks in *japonica*

To further study the different effects of the m^6^A methylome in *indica* and *japonica* rice, we sought to examine the key pathways that may be involved in rice roots shortened by Cd exposure. To eliminate the influence of rice varieties, the common genes with m^6^A modifications that were enriched in various pathways in *indica* and *japonica* under cadmium stress were detected. KEGG pathway analysis showed that these genes were involved in multiple biological pathways, including “beta-alanine metabolism”, “arginine and proline metabolism”, “pyruvate metabolism” and “histidine metabolism” (Fig. 8 A). These results indicated that cadmium treatment would affect the metabolism of various amino acids and further affect the growth and development of rice.

To further investigate the effects of m^6^A on rice growth under Cd stress, all genes with differential m^6^A peaks in *indica* and *japonica* were detected under Cd stress. Except for the above common genes, unique genes with differential m^6^A peaks in *indica* or *japonica* were enriched in various pathways. In terms of KEGG pathway analysis, “gluconeogenesis”, “plant-pathogen interaction” and autophagy-other were enriched in cv. 9311 (Fig. 8B), while “plant hormone signal transduction”, “serine” and “threonine” metabolism were enriched in cv. NIP (Fig. 8 C). Differences in unique pathways in cv. 9311 and cv. NIP may explain the phenotypic differences in *indica* and *japonica* rice under cadmium stress.

### Changes in RNA methylation-related genes in the rice response to cadmium stress

To further explore the effect of m^6^A methylation on rice growth under Cd stress, we checked whether RNA methylation-related genes were also DEGs or differentially methylated genes (DMGs) by comparing the Cd groups with the CK groups in rice.

We collected a total of 31 genes that might be related to RNA methylation in rice, including m^6^A writers, m^6^A readers and m^6^A erasers (Hu et al. 2019) (Table 1), and 17 RNA methylation-related genes were DMGs or DEGs in rice in response to Cd stress. As a result, two m^6^A writers, *LOC_Os02g45110* and *LOC_Os01g16180*, were downregulated when comparing cv. 9311_Cd with cv. 9311_CK, but they were not statistically significantly differentially expressed when comparing cv. NIP_Cd with cv. NIP_CK. The downregulation of these two genes may explain the phenomenon that the root length of cv. 9311 was significantly shortened under cadmium stress, while the variation in cv. NIP was not significant. Under Cd stress, four genes, including m^6^A writers *LOC_Os10g31030* and *LOC_Os03g35340* and m^6^A readers *LOC_Os06g46400* and *LOC_Os07g07490*, were downregulated in both cv. 9311 and cv. NIP, but there was no significant change in m^6^A methylation levels. This suggests that these genes respond to cadmium in both *indica* and *japonica* rice. The m^6^A level of the m^6^A writer *LOC_Os06g27970* was decreased when comparing cv. 9311_Cd with cv. 9311_CK, but there was no significant change in cv. NIP_Cd vs. cv. NIP_CK. The m^6^A level of the m^6^A writer *LOC_Os10g35190* was decreased in both cv. 9311 and cv. NIP under Cd stress. In contrast, the m^6^A writer *LOC_Os09g29630* was enriched with m^6^A methylation in both cv. 9311 and cv. NIP under cadmium stress. The expression levels of genes including *LOC_Os03g20180*, *LOC_Os01g48790* and *LOC_Os05g01520* were increased when comparing cv. 9311_Cd with cv. 9311_CK. As an m^6^A eraser, the m^6^A level of *LOC_Os10g02760* was decreased in both cv. 9311 and cv. NIP under Cd stress, and its expression level is decreased when comparing cv. NIP_ Cd with cv. NIP_CK, but there was no significant change. The changes in the m^6^A levels and expression levels of methylation-related genes in rice under Cd conditions may contribute to the phenotypic differences in rice after Cd treatment.

**Table 1:** RNA methylation related genes in rice. “UP” means this gene is overlapping with up hcpeaks or in up genes when comparing Cd to CKvice versa. “NDE” means this gene is not in differential hcpeaks or DE genes

RAP_id	MSU_id	gene_name	type	9311_hcpeaks	9311_DE_genes	NIP_hcpeaks	.NIP_DE_genes
Os02g0672600	LOC_Os02g45110	MTA	writers	NDE	DOWN	NDE	NDE
Os01g0267100	LOC_Os01g16180	MTB	writers	NDE	DOWN	NDE	NDE
Os03g0147700	LOC_Os03g05420	NA	writers	NDE	NDE	NDE	NDE
Os10g0447600	LOC_Os10g31030	NA	writers	NDE	DOWN	NDE	DOWN
Os06g0474200	LOC_Os06g27970	FIP37	writers	DOWN	NDE	NDE	NDE
Os03g0554900	LOC_Os03g35340	VIRLIZER	writers	NDE	DOWN	NDE	DOWN
Os10g0494500	LOC_Os10g35190	HAKAI	writers	DOWN	NDE	DOWN	NDE
Os08g0484400	LOC_Os08g37780	TRM4A	writers	NDE	NDE	NDE	NDE
Os09g0471900	LOC_Os09g29630	TRM4B	writers	UP	NDE	UP	NDE
Os01g0329800	LOC_Os01g22630	ECT11	readers	NDE	NDE	NDE	NDE
Os08g0224200	LOC_Os08g12760	ECT9	readers	NDE	NDE	NDE	NDE
Os06g0677700	LOC_Os06g46400	CPSF30	readers	NDE	DOWN	NDE	DOWN
Os03g0317000	LOC_Os03g20180	ECT7	readers	NDE	UP	NDE	NDE
Os03g0748000	LOC_Os03g53670	ECT4	readers	NDE	NDE	NDE	NDE
Os01g0679900	LOC_Os01g48790	ECT8	readers	NDE	UP	NDE	NDE
Os04g0608800	LOC_Os04g51940	ECT1	readers	NDE	UP	NDE	UP
Os08g0556000	LOC_Os08g44200	ECT5	readers	NDE	NDE	NDE	NDE
Os07g0170300	LOC_Os07g07490	ECT2	readers	NDE	DOWN	NDE	DOWN
Os04g0608900	LOC_Os04g51950	ECT6	readers	NDE	NDE	NDE	NDE
Os05g0130600	LOC_Os05g04000	ECT10	readers	NDE	UP	NDE	UP
Os05g0105600	LOC_Os05g01520	ECT3	readers	NDE	UP	NDE	NDE
Os03g0816500	LOC_Os03g60190	ALKBH1A	erasers	NDE	NDE	NDE	NDE
Os11g0488500	LOC_Os11g29690	ALKBH1B	erasers	NDE	NDE	NDE	NDE
Os06g0286310	LOC_Os06g17830	ALKBH2	erasers	NDE	NDE	NDE	NDE
Os10g0420000	LOC_Os10g28410	ALKBH6	erasers	UP	NDE	NDE	NDE
NA	LOC_Os04G51360	ALKBH8	erasers	NDE	NDE	NDE	NDE
Os11g0657200	LOC_Os11g43610	NA	erasers	NDE	NDE	NDE	NDE
Os06g0138200	LOC_Os06g04660	ALKBH9A	erasers	UP	NDE	NDE	NDE
Os05g0401500	LOC_Os05g33310	ALKBH10A	erasers	NDE	NDE	NDE	NDE
Os10g0116900	LOC_Os10g02760	ALKBH10B	erasers	DOWN	NDE	DOWN	DOWN

## Discussion

Several studies have shown that different cultivars showed different responses to Cd stress (Yan et al. 2019). In recent years, Cd has attracted much attention due to its harmful effects on plant productivity. Research has shown that cv. 9311 is a high Cd accumulating cultivar in the shoots and grain compared with cv. NIP. In contrast, cv. NIP sequesters more Cd in roots (Ueno et al. 2010). This is because OsHMA3 in NIP has a higher expression and/or functionality than that of cv. 9311(Liu et al. 2020).To date, no data have been reported about the role of the m^6^A methylome in *indica* and *japonica* rice under cadmium treatment. In the present study, we evaluated an elite *japonica* variety, cv. 9311, and an elite *indica* variety, cv. NIP, by exposing seedlings to Cd solution at 50 µM for three days. Our data showed that during the growth of rice under Cd stress, there are a large number of m^6^A methylation modifications to genes in root tissues. Therefore, understanding the genes involved these metabolic pathways might explain the differences in cadmium stress between *indica* and *japonica*. The m^6^A distribution could be influenced by exogenous stimulation. Importantly, we discovered the patterns of m^6^A distribution in cv. 9311 and cv. NIP mRNA from the CK and Cd groups. In *Arabidopsis*, m^6^A is exclusively enriched around the stop codon and start codon of genes. However, in our results, the m^6^A peak was clearly enriched in the stop codon and 3′UTR in rice.

In general, Cd is first absorbed from the soil by the roots, then it is transferred to the buds, and finally, it accumulates in the grains. The transport system plays an important role in the accumulation of Cd in rice, especially the genetic components located on the membrane (Uraguchi and Fujiwara 2013). After Cd treatment, the expression levels of 11 genes related to cadmium stress in cv. 9311 and cv. NIP were increased, and these genes, including *OsHMA4*, *PEZ1*, *OsHsfA4a*, *OsPDR8*, *OsMAPK2*, *OsABCG43*, *OsHMA9*, and *OsMSRMK2*, were not significantly m^6^A methylated (Supplementary Table S3). We measured the Cd concentration of four samples to support the phenotypic observations. The Cd concentrations of cv. 9311_CK, cv. 9311_Cd, cv. NIP_CK and cv. NIP_Cd were 1.11, 672.84, 0.94, and 1234.81 mg/kg (Supplementary Table S5), respectively. In cv. 9311, the expression level and methylation level of two genes, *OsHIR1* and *OsNramp6*, were increased. *OsHIR1*, a RING E3 ligase gene induced by heavy metals in rice, is located on the cell membrane and can control cadmium transport (Lim et al. 2014). Several metal ions such as Zn^2+^, Mn^2+^, Fe^2+^, and Cd^2+^ have been shown to be transported *via* NRAMP transporter proteins such as *OsNramp6* in rice (Mani et al. 2018). In addition, the expression level of *OsZIP1* was increased. *OsZIP1* is abundantly expressed in roots throughout the life span of the plant and is sufficiently induced by excess cadmium (Liu et al. 2019). In contrast, the methylation level of *OsHMA3* was decreased in cv. 9311 under Cd stress; this gene isolates Cd^2+^ by transporting it to the vacuole, reducing Cd^2+^ transport to the ground and thus reducing cadmium toxicity (Sasaki et al. 2014). This may be the reason for the low accumulation of Cd in cv. 9311. In cv. NIP, the expression levels of *OsLCD* and *OsCDT1* were increased under Cd stress. *OsLCD* is involved in Cd partitioning in rice, and the *lcd* mutant showed tolerance to Cd on agar plates and in hydroponic culture during early plant development (Shimo et al. 2011). Constitutive expression of *OsCDT1* confers cadmium tolerance to transgenic *A. thaliana* plants by lowering the accumulation of Cd in the cells. The changes in these genes further explained the phenotypic changes of the two rice varieties under Cd stress.

Based on the combined analysis of the transcriptome and differentially enriched m^6^A peaks in cv. 9311_Cd vs. cv. 9311_CK and cv. NIP _ Cd vs. cv. NIP_CK, nine differentially expressed genes containing m^6^A modification, which were related to root growth in rice according to previous research, were screened (Meng et al. 2019). In cv. 9311_Cd vs. cv. 9311_Ck, we found that five genes, *OsGatB*, *OsNAL1*, *OsFH1*, *OsGLU3* and *OsABIL2*, which control root growth in rice, overlapped with differentially enriched m^6^A peaks. For example, *OsGatB* may promote primary root growth by maintaining mitochondrial structure and function to facilitate cell division and elongation in the root tip (Qin et al. 2016). *OsNAL1* encodes a putative trypsin-like serine/cysteine protease that affects auxin transport (Fujita et al. 2013). *OsFH1* was also found to regulate rice root hair elongation (Huang et al. 2013). *OsGLU3* encodes a putative membrane-bound endo-1,4-β-glucanase, which is necessary for root elongation in rice (Zhang et al. 2012). These genes play an active role in rice root growth; their m^6^A levels were downregulated, and their expression was downregulated after cadmium treatment in cv. 9311. However, the m^6^A level of *OsABIL2* was downregulated, but the gene expression was upregulated in this study. Plants overexpressing *OsABIL2* had attenuated ABA signalling and shorter root hairs (Wang et al. 2017), which means that this gene has a negative regulatory effect on rice growth. The results were consistent with the phenotype of cv. 9311 rice treated with Cd. In cv. NIP_Cd vs. cv. NIP_CK, we found that three genes, *OsCHR4*, *OsSLL1* and *OsSNDP1*, overlapped with differentially modified peaks. *OsSLL1*, encoding a stearoyl acyl carrier protein from the fatty acid desaturase family, affects overall fatty acid desaturation (Shelley et al. 2017). *OsSNDP1*, encoding a phosphatidylinositol transfer protein (PITP), promotes root hair elongation via phospholipid signalling and metabolism (Huang et al. 2013). These two genes have a negative regulatory effect on rice growth, and their m^6^A levels were decreased, but the expression levels of these genes were increased. *OsCHR4* plays a role in crown root development through the auxin signalling pathway (Zhao et al. 2012). The m^6^A methylation level of *OsCHR4* was upregulated, but the expression level was downregulated in cv. NIP_ Cd vs. cv. NIP_CK. *OsAIM1* is also required for root growth in rice by promoting reactive oxygen species (ROS) accumulation (Xu et al. 2017). This gene was found in both cv. 9311 and cv. NIP, which indicates that this gene may be a common methylation modification gene that responds to Cd stress in rice. The m^6^A methylation level of *OsAIM1* was decreased, but the expression level of was upregulated in both cv. NIP_ Cd vs. cv. NIP_CK and cv. 9311_Cd vs. cv. 9311_CK. Our study suggests that the number and extent of m^6^A modifications on the transcripts of Cd-resistance genes may be important factors for determining and assessing the Cd tolerance of crops.

## Methods

### Cultivation and treatment of rice seedlings

Seeds of the *indica* rice cv. 9311 and the *japonica* rice cv. NIP were germinated. Each type of rice seedling was divided into two groups. Each group was repeated three times, and plants were exposed to 0 or 50 µM CdCl_2_ in hydroponic culture for three days. The seedlings were grown in a growth chamber at 28 °C under a 16 h light/8 h dark cycle with a light period from 6:00 AM to 10:00 PM for five days; the distilled water with or without CdCl_2_ was changed every day. After treatment for three days, rice roots from the CK and Cd groups were harvested, snap frozen in liquid nitrogen and then refrigerated at − 80 °C for RNA isolation and sequencing.

### RNA isolation and library construction

Total RNA was extracted using TRIzol reagent (Invitrogen, CA, USA) following the manufacturer’s procedure. The total RNA quality and quantity were analysed using a Bioanalyzer 2100 and RNA 6000 Nano Lab Chip Kit (Agilent, CA, USA). Only RNAs with a RIN number > 7.0 were used for library construction. Approximately 200 µg of total RNA was subjected to isolation of poly(A) mRNA with poly-T oligo-attached magnetic beads (Invitrogen). Following purification, the poly(A) mRNA fractions were fragmented into ~ 100-nt-long oligonucleotides using divalent cations under elevated temperature. Then, the cleaved RNA fragments were incubated for 2 h at 4 °C with an m6A-specific antibody (No. 202,003, Synaptic Systems, Germany) in IP buffer (50 mM Tris-HCl, 750 mM NaCl and 0.5 % Igepal CA-630) supplemented with BSA (0.5 µg µl − 1). The mixture was then incubated with protein-A beads and eluted with elution buffer (1 × IP buffer and 6.7 mM m^6^A). Eluted RNA was precipitated with 75 % ethanol. Eluted m^6^A-containing fragments (IP) and untreated input control fragments were converted to the final cDNA library via strand-specific library preparation by the dUTP method. The average insert size for the paired-end libraries was ~ 100 ± 50 bp. Then, we performed paired-end 2 × 150 bp sequencing on an Illumina NovaSeq™ 6000 platform at LC-BIO Biotech Ltd. (Hangzhou, China) following the vendor’s recommended protocol.

After exposure of rice seedlings of cv. 9311 and cv. NIP to Cd, extensive phenotypic variations were observed for seedling length under Cd stress and control conditions. After the experiment was carried out continuously for 7 days, rice roots were collected for the measurement of their lengths.

### m^6^A sequencing

Rice roots from CK- and Cd^2+^-stressed rice plants were collected to extract the total RNA. Three biological replicates of m^6^A RIP sequencing were performed for the four rice samples. MACS_2_ was used to call m^6^A peaks with strict standards (error detection rate (FDR) < 0.05, *p*-value < 0.01, fold change (FC) > 2). Homer software was used to identify the new motifs in the m^6^A peaks and obtain their position weight matrices and precise motif regions. We assigned all modification sites to genic regions, including the CDS, 3’UTR, 5’ UTR, intron and exon region. The genic regions were separated into five regions: (1) 5’ UTR, in which 100 bp close to start codon was removed; (2) start codon region, which is a 200 bp long region extracted from the 5’ UTR and CDS regions centred at the start codon; (3) CDS region, in which the 100 bp region after the start codon and 100 bp region before the stop codon were removed; (4) stop codon region, which is a 200 bp long region extracted from the CDS and 3’ UTR regions centred at stop codon; and (5) intron region, which includes all introns of the gene. Unique peaks (unipeak) were assigned to one of the 5 genic regions described above based on genomic coordinates with a minimum overlap of 100 bp. Peaks unable to be assigned to one of 5 genic regions were classified as intergenic. Then, the differentially expressed genes were identified using edgeR (Nikolayeva et al. 2014). Gene Ontology (GO) enrichment analysis was performed using AgriGOv2 (Tian et al. 2017).

### Processing of raw data

Raw sequencing data were analysed using fastQC (v0.11.7). The R package “ngsReports” was used to summarize fastQC reports. Low-quality and adaptor sequences were trimmed from raw reads using trim_galore (v0.4.4) with the following parameters: --stringency 6 -a-aAAGTCGGAGGCCAAGCGGTCTTAGGAAGACAA-a2AAGTCGGATCGTAGCCATGTCGTTCTGTGAGCCAAGGAGTTG --fastqc --paired.

### Genome mapping

Clean reads were mapped to the rice reference genome IRGSP-1.0 (https://plants.ensembl.org/Oryza_sativa/Info/Index) with gene annotation Release 48 (ftp://ftp.ensemblgenomes.org/pub/plants/release-48/gff3/oryza_sativa) using STAR (v2.7.6a) with the following parameters: --outFilterMismatchNmax 6 --outFilterMismatchNoverLmax 0.03 --quantMode.

### Comparison of peaks in different groups

Only unipeaks that are present in at least two out of three biological replicates (minimum overlap was 100 bp with identified peaks in each biological replicate) are considered high confident peaks (named hcpeak) in this study.

### Quantitative real-time PCR (qRT-PCR) validation

To validate the RNA-seq results, different expression patterns of several genes were confirmed by quantitative real-time RT-PCR (qRT-PCR). For qRT-PCR, 1 µg of total RNA was used to synthesize cDNA using the PrimeScriptTM RT reagent Kit (Perfect Real Time) (TaKaRa). qRT-PCR was carried out using SYBR® Premix Ex Taq II (Tli RNaseH Plus; TAKARA BIO Inc., Shiga, Japan) and determined in a LightCycler 480 (Roche, Basel, Switzerland) according to the manufacturer’s instructions. The qRT-PCRs were amplified at 95 °C for 30 s, followed by 40 cycles of 95 °C for 5 s, 55 °C for 30 s and 72 °C for 30 s. All reactions were performed with three independent biological replicates for each sample, and three technical replicates for each biological replicate were analysed. The relative gene expression was calculated by ABI7500 Real-Time PCR System software using the 2 ^−∆∆Ct^ method. The primers used for real-time qPCR are listed in Supplementary Table S4.

## Conclusions

The study reported effects of Cd stress on m^6^A mRNA methylation and related gene expression in rice roots. Using the MERIP-seq technique, we found a large number of changes in m^6^A signaling in Cd-exposed rice roots. After GO and KEGG analysis, the enrichment and pathways of many genes were analyzed. These differences may be physiologically related to the observed variations in the Cd tolerance of different plant species. This study is helpful to understand the relationship between m^6^A modification and Cd stress response in rice.

## Supplementary Information

**Additional file 1:**

## Data Availability

The data sets supporting the results of this article are included within the article and its additional files.

## Abbreviations

m^6^A: N^6^-methyladenosine
Cd: Cadmium
CK: Control
cv. NIP: Nipponbare
m^6^A peak: m^6^A-modified nucleotide position on mRNAs
IP: Immunoprecipitation
non-IP control: input
hcpeaks: High confident peaks
GO: Gene ontology
DMPs: Differential methylated peaks
DEGs: Differentially expressed genes
DMGs: Differentially methylated genes
ROS: Reactive oxygen species
METTL3: Methyltransferase-like 3
METTL14: Methyltransferase-like 14
RBM15: RNA binding motif protein 15
HAKAI: Cbl photo oncogene like 1
ZC3H13: Zinc finger CCCH domain-containing protein 13
MTA: mRNA adenosine methylase
FIP37: FKBP12 interacting protein 37
WTAP: Wilms’ tumour1-associating protein
YTH: for YT521-B Homology
ECT2/3: EVOLUTIONARILY CONSERVED C-TERMINAL REGION2/3

## References

[CR1] Anderson SJ, Kramer MC, Gosai SJ, Yu X, Vandivier LE, Nelson ADL, Anderson ZD, Beilstein MA, Fray RG, Lyons E, Gregory BD (2018). N^6^-Methyladenosine inhibits local ribonucleolytic cleavage to stabilize mRNAs in Arabidopsis. Cell Rep.

[CR2] Arribas-Hernández L, Bressendorff S, Hansen MH, Poulsen C, Erdmann S, Brodersen P (2018). An m^6^A-YTH Module Controls Developmental Timing and Morphogenesis in Arabidopsis. Plant Cell.

[CR3] Cao ZZ, Lin XY, Yang YJ, Guan MY, Xu P, Chen MX (2019). Gene identification and transcriptome analysis of low cadmium accumulation rice mutant (lcd1) in response to cadmium stress using MutMap and RNA-sEq. BMC Plant Biol.

[CR4] De Jesus DF, Zhang Z, Kahraman S, Brown NK, Chen M, Hu J, Gupta MK, He C, Kulkarni RN (2019). m^6^A mRNA methylation regulates human β-Cell biology in physiological states and in Type 2 Diabetes. Nat Metab.

[CR5] Duan HC, Wei LH, Zhang C, Wang Y, Chen L, Lu Z, Chen PR, He C, Jia G (2017). ALKBH10B is an RNA N^6^-Methyladenosine demethylase affecting Arabidopsis floral transition. Plant Cell.

[CR6] Feng SJ, Liu XS, Tao H, Tan SK, Chu SS, Oono Y, Zhang XD, Chen J, Yang ZM (2016). Variation of DNA methylation patterns associated with gene expression in rice *(Oryza sativa*) exposed to cadmium. Plant Cell Environ.

[CR7] Feng SJ, Liu XS, Ma LY, Khan IU, Rono JK, Yang ZM (2020) Identification of epigenetic mechanisms in paddy crop associated with lowering environmentally related cadmium risks to food safety. Environ Pollut 256: 113464.1-113464.12 10.1016/j.envpol.2019.113464 31677869

[CR8] Fujita D, Trijatmiko KR, Tagle AG, Sapasap MV, Koide Y, Sasaki K, Tsakirpaloglou N, Gannaban RB, Nishimura T, Yanagihara S, Fukuta Y, Koshiba T, Slamet-Loedin IH, Ishimaru T, Kobayashi N (2013). NAL1 allele from a rice landrace greatly increases yield in modern indica cultivars. Proc Natl Acad Sci USA.

[CR9] Hu J, Manduzio S, Kang H (2019). Epitranscriptomic RNA methylation in plant development and abiotic stress responses. Front Plant Sci.

[CR10] Huang J, Kim CM, Xuan YH, Liu J, Kim TH, Kim BK, Han CD (2013). Formin homology 1 (OsFH1) regulates root-hair elongation in rice (Oryza sativa). Planta.

[CR11] Huang J, Kim CM, Xuan YH, Park SJ, Piao HL, Je BI, Liu J, Kim TH, Kim BK, Han CD (2013). OsSNDP1, a Secs.&nbsp;14-nodulin domain-containing protein, plays a critical role in root hair elongation in rice. Plant Mol Biol.

[CR12] Lence T, Akhtar J, Bayer M, Schmid K, Spindler L, Ho CH, Kreim N, Andrade-Navarro MA, Poeck B, Helm M, Roignant JY (2016). m^6^A modulates neuronal functions and sex determination in Drosophila. Nature.

[CR13] Lim SD, Hwang JG, Han AR, Park YC, Lee C, Ok YS, Jang CS (2014). Positive regulation of rice RING E3 ligase OsHIR1 in arsenic and cadmium uptakes. Plant Mol Biol.

[CR14] Liu XS, Feng SJ, Zhang BQ, Wang MQ, Cao HW, Rono JK, Chen X, Yang ZM (2019). OsZIP1 functions as a metal efflux transporter limiting excess zinc, copper and cadmium accumulation in rice. BMC Plant Biol.

[CR15] Liu CL, Gao ZY, Shang LG, Yang CH, Ruan BP, Zeng DL, Guo LB, Zhao FJ, Huang CF, Qian Q (2020). Natural variation in the promoter of OsHMA3 contributes to differential grain cadmium accumulation between *Indica* and *Japonica* rice. J Integr Plant Biol.

[CR16] Mani A, Sankaranarayanan K (2018). In silico analysis of natural resistance-associated macrophage protein (NRAMP) family of transporters in rice. Protein J.

[CR17] Meng F, Xiang D, Zhu J, Li Y, Mao C (2019). Molecular Mechanisms of Root Development in Rice. Rice (N Y).

[CR18] Meyer KD, Jaffrey SR (2017) Rethinking m6A Readers, Writers, and Erasers. Annual Rev Cell Deve Biol 33(1):319 10.1146/annurev-cellbio-100616-060758 PMC5963928 28759256

[CR19] Meyer KD, Saletore Y, Zumbo P, Elemento O, Mason CE, Jaffrey SR (2012). Comprehensive analysis of mRNA methylation reveals enrichment in 3’ UTRs and near stop codons. Cell.

[CR20] Meyer KD, Patil DP, Zhou J, Zinoviev A, Skabkin MA, Elemento O, Pestova TV, Qian SB, Jaffrey SR (2015). 5’ UTR m (6)A promotes Cap-Independent translation. Cell.

[CR21] Miao Z, Zhang T, Qi Y, Song J, Han Z, Ma C (2020). Evolution of the RNA N^6^-methyladenosine methylome mediated by genomic duplication. Plant Physiol.

[CR22] Nikolayeva O, Robinson MD (2014). EdgeR for differential RNA-seq and ChIP-seq analysis: an application to stem cell biology. Methods Mol Biol.

[CR23] Parker MT, Knop K, Simpson GG (2020). Making a mark: the role of RNA modifications in plant biology. Biochem.

[CR24] Qin C, Cheng L, Zhang H, He M, Shen J, Zhang Y, Wu P (2016). OsGatB, the subunit of tRNA-Dependent amidotransferase, is required for primary root development in rice. Front Plant Sci.

[CR25] Santos RWD, Schmidt Éder C, Martins RDP, Latini A, Maraschin M, Horta PA, Bouzon ZL (2012). Effects of cadmium on growth, photosynthetic pigments, photosynthetic performance, biochemical parameters and structure of chloroplasts in the agarophyte gracilaria domingensis (Rhodophyta, Gracilariales). American Journal of Plant Sciences.

[CR26] Sasaki A, Yamaji N, Ma JF (2014). Overexpression of OsHMA3 enhances Cd tolerance and expression of Zn transporter genes in rice. J Exp Bot.

[CR27] Shelley IJ, Nishiuchi S, Shibata K, Inukai Y (2017). SLL1, which encodes a member of the stearoyl-acyl carrier protein fatty acid desaturase family, is involved in cell elongation in lateral roots via regulation of fatty acid content in rice. Plant Sci.

[CR28] Shen L, Liang Z, Gu X, Chen Y, Teo ZW, Hou X, Cai WM, Dedon PC, Liu L, Yu H (2016). N (6)-Methyladenosine RNA modification regulates shoot stem cell fate in Arabidopsis. Dev Cell.

[CR29] Shimo H, Ishimaru Y, An G, Yamakawa T, Nakanishi H, Nishizawa NK (2011). Low cadmium (LCD), a novel gene related to cadmium tolerance and accumulation in rice. J Exp Bot.

[CR30] Tan M, Cheng D, Yang Y, Zhang G, Qin M, Chen J, Chen Y, Jiang M (2017). Co-expression network analysis of the transcriptomes of rice roots exposed to various cadmium stresses reveals universal cadmium-responsive genes. BMC Plant Biol.

[CR31] Tian T, Liu Y, Yan H, You Q, Yi X, Du Z, Xu W, Su Z (2017). agriGO v2.0: a GO analysis toolkit for the agricultural community. Nucleic Acids Res.

[CR32] Ueno D, Yamaji N, Kono I, Huang CF, Ando T, Yano M, Ma JF (2010). Gene limiting cadmium accumulation in rice. Proc Natl Acad Sci USA.

[CR33] Uraguchi S, Fujiwara T (2013). Rice breaks ground for cadmium-free cereals. Curr Opin Plant Biol.

[CR34] Wang T, Li C, Wu Z, Jia Y, Wang H, Sun S, Mao C, Wang X (2017). Abscisic acid regulates auxin homeostasis in rice root tips to promote root hair elongation. Front Plant Sci.

[CR35] Wei LH, Song PZ, Wang Y, Lu ZK, Tang Q, Yu Q, Xiao Y, Zhang X, Duan HC, Jia GF (2018). The m^6^A reader ECT2 controls trichome morphology by affecting mRNA stability in Arabidopsis. Plant Cell.

[CR36] Wu B, Jiang X, Huang YP, Ying XL, Zhang HQ, Liu BX, Li Z, Qi DF, Ji WD, Cai XM (2021) Integrated analysis of mRNA-m^6^A-protein profiles reveals novel insights into the mechanisms for cadmium-induced urothelial transformation. Biomarkers 2021:1–23 10.1080/1354750X.2021.1913513 33830842

[CR37] Xin C, Chi J, Zhao Y, He Y, Guo J. Cadmium stress alters cytosine methylation status and expression of a select set of genes in Nicotiana benthamiana. Plant Sci 2019 284:16–24 10.1016/j.plantsci.2019.03.021 31084868

[CR38] Xu L, Zhao H, Ruan W, Deng M, Wang F, Peng J, Luo J, Chen Z, Yi K (2017). Abnormal inflorescence meristem1 functions in salicylic acid biosynthesis to maintain proper reactive oxygen species levels for root meristem activity in rice. Plant Cell.

[CR39] Yan H, Xu W, Xie J, Gao Y, Wu L, Sun L, Feng L, Chen X, Zhang T, Dai C, Li T, Lin X, Zhang Z, Wang X, Li F, Zhu X, Li J, Li Z, Chen C, Ma M, Zhang H, He Z (2019). Variation of a major facilitator superfamily gene contributes to differential cadmium accumulation between rice subspecies. Nat Commun.

[CR40] Yang Y, Hsu PJ, Chen YS, Yang YG (2018). Dynamic transcriptomic m^6^A decoration: writers, erasers, readers and functions in RNA metabolism. Cell Res.

[CR41] Yao Y, Bi Z, Wu R, Zhao Y, Liu Y, Liu Q, Wang Y, Wang X (2019). METTL3 inhibits BMSC adipogenic differentiation by targeting the JAK1/STAT5/C/EBPβ pathway via an m6A-YTHDF2-dependent manner. FASEB J.

[CR42] Yue H, Nie X, Yan Z, Weining S (2019). N^6^-methyladenosine regulatory machinery in plants: composition, function and evolution. Plant Biotechnol J.

[CR43] Zhang JW, Xu L, Wu YR, Chen XA, Liu Y, Zhu SH, Ding WN, Wu P, Yi KK (2012). OsGLU3, a putative membrane-bound endo-1,4-beta-glucanase, is required for root cell elongation and division in rice (Oryza sativa L.). Mol Plant.

[CR44] Zhang F, Zhang YC, Liao JY, Yu Y, Zhou YF, Feng YZ, Yang YW, Lei MQ, Bai M, Wu H, Chen YQ (2019). The subunit of RNA N^6^-methyladenosine methyltransferase OsFIP regulates early degeneration of microspores in rice. PLoS Genet.

[CR45] Zhao C, Xu J, Chen Y, Mao C, Zhang S, Bai Y, Jiang D, Wu P (2012). Molecular cloning and characterization of OsCHR4, a rice chromatin-remodeling factor required for early chloroplast development in adaxial mesophyll. Planta.

[CR46] Zheng H, Sun X, Li J, Song Y, Song J, Wang F, Liu L, Zhang X, Sui N (2021). Analysis of N^6^-methyladenosine reveals a new important mechanism regulating the salt tolerance of sweet sorghum. Plant Sci.

