# Supplementary material for: Coordination of m6A mRNA methylation and gene transcriptome in rice response to cadmium stress
Source: Rice (N Y). 2021 Jul 5;14:62. doi: 10.1186/s12284-021-00502-y (PMC8257850; doi:10.1186/s12284-021-00502-y)
Supplement: Supplementary file 1 — Additional file 1: [file 12284_2021_502_MOESM1_ESM.docx]

**Appendix A. Supplementary data**

**Supplementary Figures and Tables Captions**

**Supplementary Fig. S1.** Shoots length of cv. 9311 and cv. NIP under CK (control) and Cd (cadmium)groups. **(A)** and **(B)** Comparison of root length, in 3-day-old seedlings of cv. 9311 and cv. NIP under under control condition and Cd stress conditions, respectively. Data are presented as means ± SE. n = 15. Statistical analysis was conducted using the Student’s t-test. *, P-value < 0.05; **, P-value < 0.01; ***, Pvalue < 0.001.

**
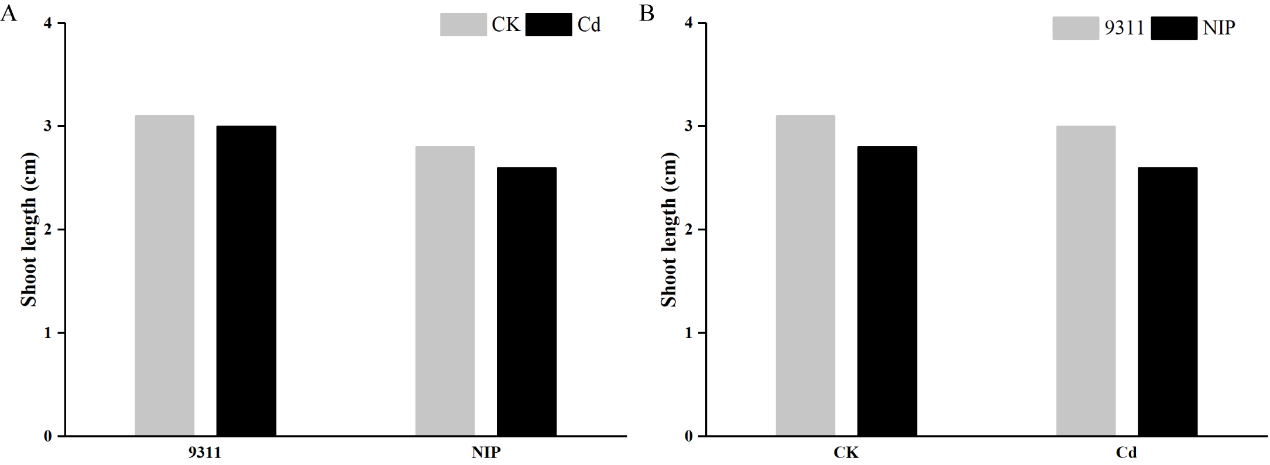
**

**Supplementary Fig. S2.** relative mRNA levels validated by qRT-PCR


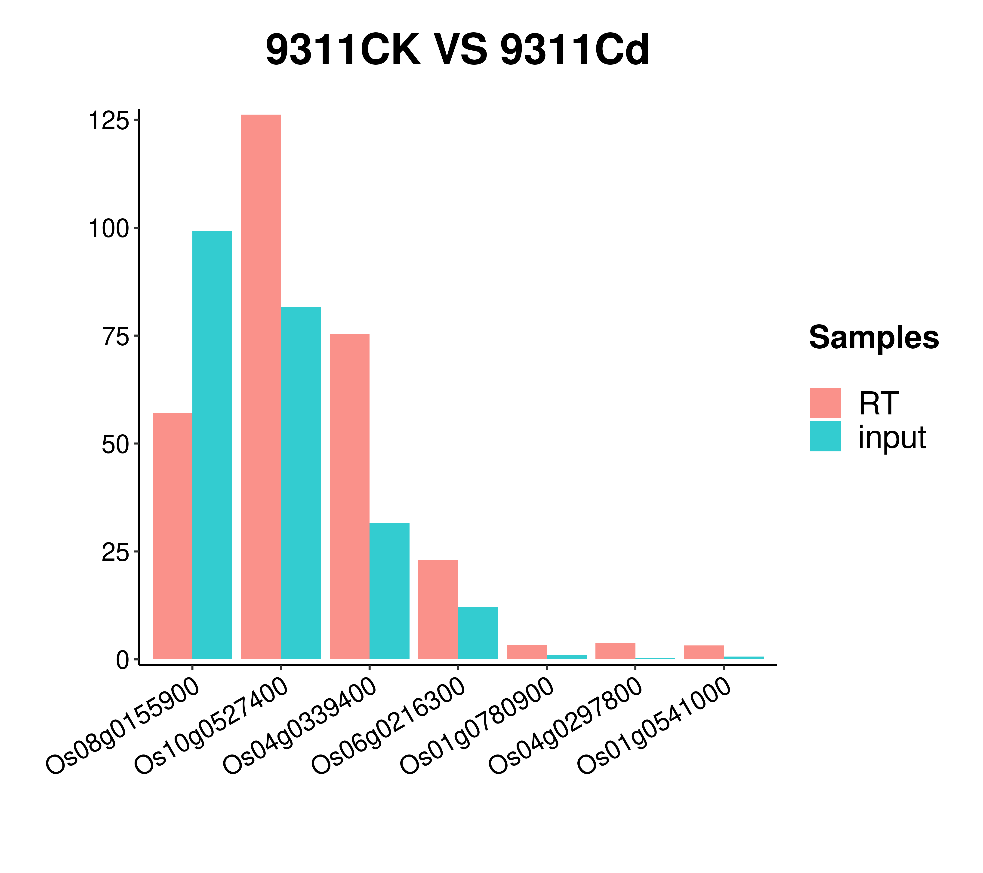


**Supplementary Fig. S3.** Distribution of m^6^A peaks in four experimental conditions. (A) Fractions, (B), (C)and (D) relative enrichment of m^6^A peaks in six nonoverlapping transcript segments: 5′ UTRs, start codons, CDS, stop codons, TSS and 3′ UTRs.


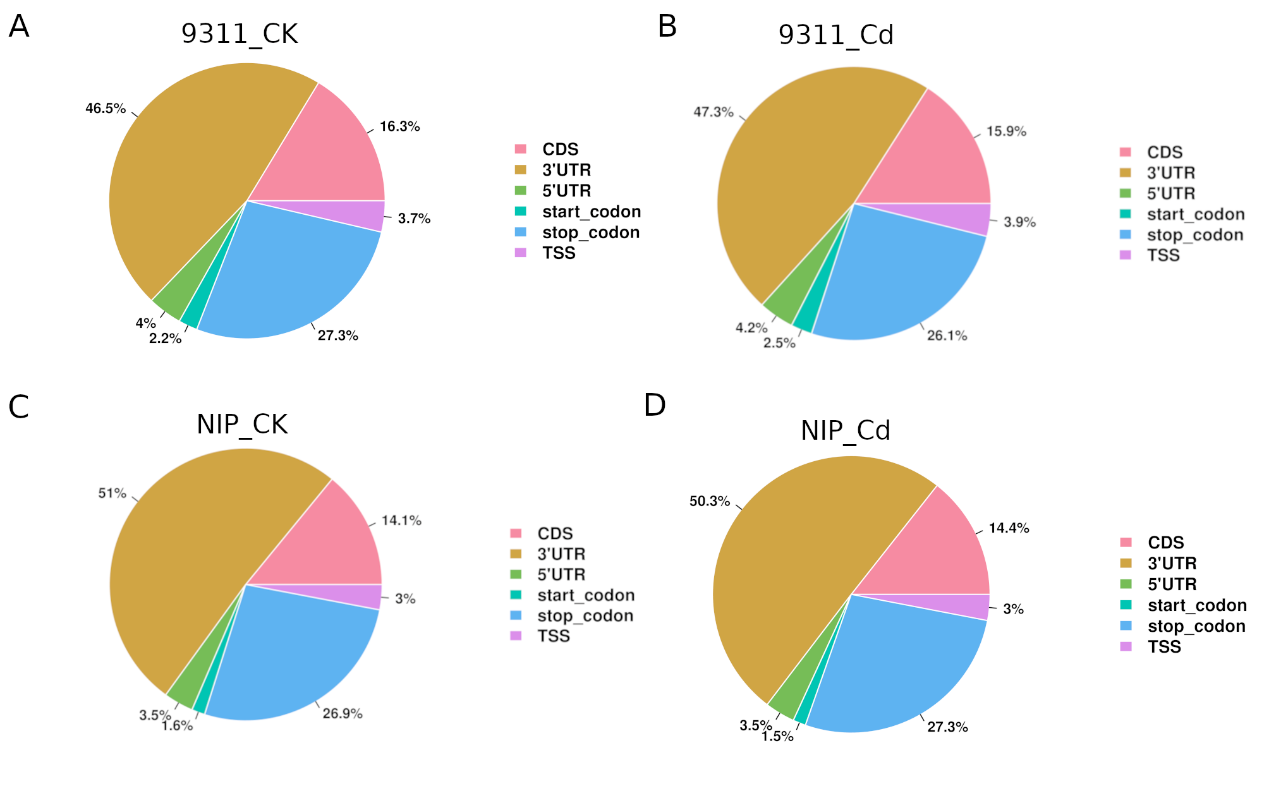


**Supplementary Fig. S4** Compare the differential hcpeaks and DE genes.

**(A)** comparison of genes overlapping with differential hcpeaks and DE genes in cv. 9311_Cd *vs* cv. NIP_ Cd **(B)** comparison of genes overlapping with differential hcpeaks and DE genes in cv. 9311_ck *vs* cv. NIP_ ck.


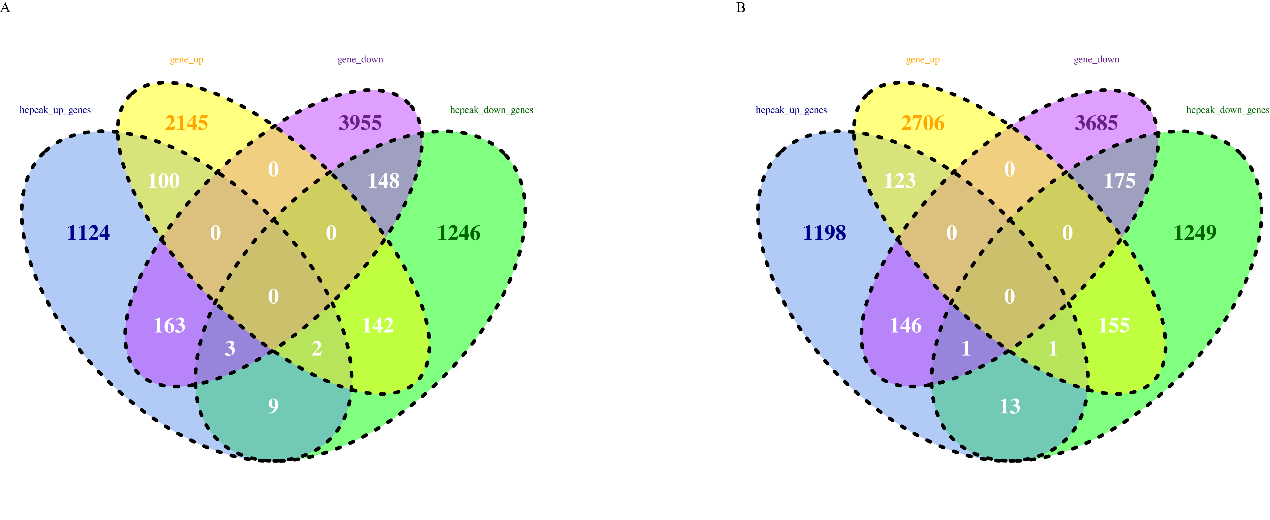


**Supplementary Table S1.** Summary of reads quality control.

| Sample_ID | Raw_Reads | Valid_Reads | Valid% | Q20% | Q30% |
| --- | --- | --- | --- | --- | --- |
| 9311_Cd1_IP | 67259878 | 62242012 | 84.30 | 98.65 | 95.45 |
| 9311_Cd 2_IP | 64873816 | 59531924 | 83.49 | 98.69 | 95.51 |
| 9311_Cd 3_IP | 70384052 | 65917902 | 84.95 | 98.63 | 95.38 |
| 9311_CK1_IP | 69846630 | 63389786 | 82.76 | 98.71 | 95.56 |
| 9311_CK2_IP | 63965380 | 58954284 | 84.72 | 98.67 | 95.47 |
| 9311_CK3_IP | 57909012 | 55456780 | 87.74 | 98.59 | 95.30 |
| NIP_Cd1_IP | 64771488 | 61246300 | 86.37 | 98.61 | 95.37 |
| NIP_Cd2_IP | 72752356 | 59739746 | 75.07 | 98.62 | 95.40 |
| NIP_Cd3_IP | 72721782 | 63112304 | 79.30 | 98.66 | 95.48 |
| NIP_CK1_IP | 61690248 | 57677808 | 85.44 | 98.70 | 95.53 |
| NIP_CK2_IP | 61453436 | 55806788 | 83.14 | 98.64 | 95.41 |
| NIP_CK3_IP | 72714228 | 67849584 | 85.11 | 98.68 | 95.55 |
| 9311_Cd1_input | 75479226 | 73010726 | 88.88 | 97.24 | 92.01 |
| 9311_Cd2_input | 74172300 | 71837780 | 88.67 | 97.23 | 91.98 |
| 9311_Cd3_input | 72687242 | 70624020 | 89.16 | 97.10 | 91.76 |
| 9311_CK1_input | 74013554 | 71818434 | 88.74 | 97.36 | 92.25 |
| 9311_CK2_input | 76478032 | 74401868 | 89.39 | 97.26 | 92.08 |
| 9311_CK3_input | 74008778 | 71776102 | 88.69 | 97.26 | 92.04 |
| NIP_Cd1_input | 72140082 | 70002664 | 88.72 | 97.16 | 91.88 |
| NIP_Cd2_input | 76873534 | 74690828 | 88.50 | 97.20 | 91.96 |
| NIP_Cd3_input | 63250858 | 60276994 | 86.94 | 98.50 | 95.28 |
| NIP_CK1_input | 59283270 | 57255310 | 88.35 | 98.52 | 95.32 |
| NIP_CK2_input | 60959830 | 58114726 | 86.88 | 98.49 | 95.26 |
| NIP_CK3_input | 65929070 | 63917780 | 88.63 | 98.54 | 95.39 |

**Supplementary Table S2.** Summary of the presence of unipeaks in biological replictes of each group

| Presence in replicates | 9311_Cd | 9311_CK | NIP_Cd | NIP_CK |
| --- | --- | --- | --- | --- |
| 0 | 2537 | 2890 | 2385 | 3638 |
| 1 | 1333 | 1713 | 1751 | 1391 |
| 2 | 1070 | 1304 | 1411 | 1182 |
| 3 | 7902 | 6935 | 7295 | 6631 |

**Supplementary Table S3.** Cadmlation related genes in rice. “UP” indicates that this gene is overlapping with significantly enriched m^6^A methylation peaks in the Cd treated group compared to the control group, and vice versa. “NDE” indicates that this gene is not overlapping with any significantly differential m^6^A peaks when comparing the Cd treated group with the control group.

| gene | RAP_id | MSU | 9311_hcpeaks | 9311_DE_genes | NIP_hcpeaks | NIP_DE_genes |
| --- | --- | --- | --- | --- | --- | --- |
| OsNAAT1 | Os02g0306401 | LOC_Os02g20360 | NDE | NDE | NDE | NDE |
| OsHMA4 | Os02g0196600 | LOC_Os02g10290 | NDE | UP | NDE | UP |
| PEZ1 | Os03g0571900 | LOC_Os03g37490 | NDE | UP | NDE | UP |
| OsZIP3 | Os04g0613000 | LOC_Os04g52310 | NDE | NDE | NDE | NDE |
| OsHsfA4a | Os01g0749300 | LOC_Os01g54550 | NDE | UP | NDE | UP |
| OsPDR8 | Os01g0609900 | LOC_Os01g42410 | NDE | UP | NDE | UP |
| OSMTI-1b | Os03g0288000 | LOC_Os03g17870 | NDE | UP | NDE | NDE |
| OsPCR1 | Os10g0112100 | LOC_Os10g02300 | NDE | NDE | NDE | NDE |
| rgMT | Os11g0704500 | LOC_Os11g47809 | NDE | NDE | NDE | NDE |
| RCS1 | Os12g0625000 | LOC_Os12g42980 | NDE | UP | NDE | UP |
| OsPCS2 | Os06g0102300 | LOC_Os06g01260 | DOWN | NDE | DOWN | NDE |
| OsLCD | Os01g0956700 | LOC_Os01g72670 | NDE | NDE | NDE | UP |
| OsCDT3 | Os01g0178300 | LOC_Os01g08300 | NDE | NDE | NDE | NDE |
| Ospdr9 | Os01g0609300 | LOC_Os01g42380 | NDE | UP | NDE | UP |
| OsWJUMK1 | Os01g0665200 | LOC_Os01g47530 | DOWN | UP | NDE | UP |
| OsZIP1 | Os01g0972200 | LOC_Os01g74110 | NDE | UP | NDE | NDE |
| OsAUX1 | Os01g0856500 | LOC_Os01g63770 | NDE | NDE | NDE | NDE |
| OsCDT4 | Os02g0208500 | LOC_Os02g11770 | NDE | DOWN | NDE | DOWN |
| OsYSL2 | Os02g0649900 | LOC_Os02g43370 | NDE | NDE | NDE | NDE |
| OsCDT1 | Os03g0656500 | LOC_Os03g45370 | NDE | NDE | NDE | UP |
| OsIRT1 | Os03g0667500 | LOC_Os03g46470 | NDE | NDE | NDE | NDE |
| OsIRT2 | Os03g0667300 | LOC_Os03g46454 | NDE | NDE | NDE | NDE |
| OsMSRMK2 | Os03g0285800 | LOC_Os03g17700 | NDE | UP | NDE | UP |
| OsCDT5 | Os05g0178300 | LOC_Os05g08554 | NDE | NDE | NDE | NDE |
| OsMTP1 | Os05g0128400 | LOC_Os05g03780 | NDE | NDE | NDE | NDE |
| OsHMA2 | Os06g0700700 | LOC_Os06g48720 | NDE | NDE | NDE | NDE |
| OsHMA9 | Os06g0665800 | LOC_Os06g45500 | NDE | UP | NDE | UP |
| OsLCT1 | None | LOC_Os06g38120 | NDE | NDE | NDE | NDE |
| OsMSRMK3 | Os06g0699400 | LOC_Os06g48590 | NDE | UP | NDE | NDE |
| OsABCG43 | Os07g0522500 | LOC_Os07g33780 | NDE | UP | NDE | UP |
| OsHMA3 | Os07g0232900 | LOC_Os07g12900 | DOWN | NDE | NDE | NDE |
| OsNramp1 | Os07g0258400 | LOC_Os07g15460 | NDE | NDE | NDE | NDE |
| OsNramp5 | Os07g0257200 | LOC_Os07g15370 | NDE | NDE | NDE | NDE |
| OsMAPK2 | Os08g0157000 | LOC_Os08g06060 | DOWN | UP | NDE | UP |
| OsHIR1 | Os08g0398400 | LOC_Os08g30790 | UP | UP | NDE | NDE |
| OsNramp6 | Os01g0503400 | LOC_Os01g31870 | UP | UP | NDE | NDE |

**Supplemental Table S4**. Primers used in this study.

| Primers used for qPCR | | |
| --- | --- | --- |
| Name | Forward primer (5'-3') | Reverse primer (5'-3') |
| Os08g0155900 | TCTACAAGTACGGCTTCGCC | AGCTGTCGCCGTCGC |
| Os10g0527400 | AAGGGCCTGAGCTACGACTA | GGACCTTCTTGTGTACCGGG |
| Os04g0339400 | CATCGAATCGACTCCACGGT | CCACGTGAGCCCTCCTTATC |
| Os06g0216300 | TTCATGAAGGACAGCGCCAA | AAGTCGACGAACGGCGATAA |
| Os01g0780900 | GTGGGGATCCTTTGCTGAGT | AACCGTTAATCCCCATCGGC |
| Os04g0297800 | CCATCGTTGAAGGTTGGCTG | GTTCGAGCTCAACCCGAATG |
| Os01g0541000 | CGACCAGACAATCGTTTCGC | CATTTCGCTGATAGTGCGCC |

Supplementary Table S5. Cd concentration for four samples(mg/kg).

| 9311_CK | 1.11 |
| --- | --- |
| 9311_Cd | 672.84 |
| NIP_ CK | 0.94 |
| NIP_ Cd | 1234.81 |
